# A deep learning model based on whole slide images to predict disease-free survival in cutaneous melanoma patients

**DOI:** 10.1038/s41598-022-24315-1

**Published:** 2022-11-27

**Authors:** Maria Colomba Comes, Livia Fucci, Fabio Mele, Samantha Bove, Cristian Cristofaro, Ivana De Risi, Annarita Fanizzi, Martina Milella, Sabino Strippoli, Alfredo Zito, Michele Guida, Raffaella Massafra

**Affiliations:** I.R.C.C.S. Istituto Tumori “Giovanni Paolo II”, Viale Orazio Flacco 65, 70124 Bari, Italy

**Keywords:** Cancer, Cancer imaging, Skin cancer

## Abstract

The application of deep learning on whole-slide histological images (WSIs) can reveal insights for clinical and basic tumor science investigations. Finding quantitative imaging biomarkers from WSIs directly for the prediction of disease-free survival (DFS) in stage I–III melanoma patients is crucial to optimize patient management. In this study, we designed a deep learning-based model with the aim of learning prognostic biomarkers from WSIs to predict 1-year DFS in cutaneous melanoma patients. First, WSIs referred to a cohort of 43 patients (31 DF cases, 12 non-DF cases) from the Clinical Proteomic Tumor Analysis Consortium Cutaneous Melanoma (CPTAC-CM) public database were firstly annotated by our expert pathologists and then automatically split into crops, which were later employed to train and validate the proposed model using a fivefold cross-validation scheme for 5 rounds. Then, the model was further validated on WSIs related to an independent test, i.e. a validation cohort of 11 melanoma patients (8 DF cases, 3 non-DF cases), whose data were collected from Istituto Tumori ‘Giovanni Paolo II’ in Bari, Italy. The quantitative imaging biomarkers extracted by the proposed model showed prognostic power, achieving a median AUC value of 69.5% and a median accuracy of 72.7% on the public cohort of patients. These results remained comparable on the validation cohort of patients with an AUC value of 66.7% and an accuracy value of 72.7%, respectively. This work is contributing to the recently undertaken investigation on how treat features extracted from raw WSIs to fulfil prognostic tasks involving melanoma patients. The promising results make this study as a valuable basis for future research investigation on wider cohorts of patients referred to our Institute.

## Introduction

Melanoma accounts for only about 1–5% of skin cancers^1^. Among the melanoma cases, over 90% is represented from cutaneous melanoma, which is one of the most aggressive forms of skin cancer with a high mortality rate^2,3^. A prognosis improvement in cutaneous melanoma patients is crucial to better plan personalized treatments. Although more and more advanced treatments for melanoma have constantly introduced in clinical practice over the years, e.g. with the advent of checkpoint inhibitors immunotherapy and target therapy with antiBRAF/antimek drugs for patients harboring BRAF mutation^4–6^, they can cause toxicity and overtreatment, especially for early-stage patients^7^. Not less relevant, these treatments can entail significant resource expenditure due to their high costs corresponding to over $20,000 per patient per month^8^. Currently, clinical prognosis methods for the evaluation of the risk of recurrence includes multiple parameters, such as Breslow tumor thickness, ulceration, local or nodal metastasis, which are at the basis of the American Joint Committee on Cancer (AJCC) pathologic tumor stage^9,10^. Despite routinely applied in clinical practice, these clinical prognosis methods have some pitfalls. Among them, the evaluation of complete staging is performed by means of lymph node examination, that is a matter of an ongoing scientific debate due to the associated significant post-operative morbidity and/or infection^11^. Meanwhile, genomic-based tools complementing the traditional staging system are being developed in order to evaluate their prognostic power in comparison with traditional factors^12,13^. However, these tools are currently in the experimentation phase and have not yet been applied in actual clinical practice. Thus, finding more reliable and widely applicable prognostic biomarkers in melanoma patient is urgent. Within this emerging scenario, digital pathology image-based prediction models can be designed. Due to ongoing developments in technology, e.g. cloud storage systems and computer processing powers, whole slide images (WSIs), which refers to digital slides, have become the predominant imaging modality in pathology departments across the world^14^. In recent years, artificial intelligence and its deep learning branch based on Convolutional Neural Networks (CNNs) have shown potential in solving challenging imaging problems related to the medical field^15–17^. Thanks to the extraction of a huge number of image characteristics, which are naked to human eyes, correlations between image patterns and a pre-defined outcome can be recognized. The accomplishment of a specific task can be achieved either by defining an ad hoc CNNs based on a huge amount of imaging data^18^ or by using the so-called transfer learning technique based on relatively small-size datasets^19,20^. While ad hoc CNNs are trained to both extract features and make predictions, transfer learning technique allows to apply CNNs pre-trained on thousands of images as feature extractors on images under study, and then, to use a standard machine learning classifier to make prediction. Some research studies have extensively investigated the role of deep learning and in particular of CNNs in the fulfilment of diagnostic tasks related to melanoma. As example, classification of histopathological melanoma images has been performed by means of deep learning methods^21,22^. Conversely, the investigation of prognostic tasks based on WSI analysis via deep learning techniques is at the beginning stages. The sentinel lymph node status, anti-PD-L1 response as well as visceral recurrence and death in melanoma patients have been recently probed through the application of deep learning on WSIs images^23–25^. Due to the demonstrated relevance of the deep learning application on WSIs to fulfill prognostic tasks involving melanoma patients, further investigations need to be performed. In this study, we propose a deep learning model which makes use of features extracted by transfer learning to predict 1-year disease-free survival in patients with cutaneous melanoma. Whole slide images referred to a cohort of 43 patients from Clinical Proteomic Tumor Analysis Consortium Cutaneous Melanoma (CPTAC-CM) public database were firstly analyzed to design the predictive model. Then, the model was validated on independent test, i.e. a validation cohort of 11 cutaneous melanoma patients referred to our Institute. This study represents the first effort of the transfer learning use to predict disease-free survival in cutaneous melanoma patients by means of a direct analysis of WSIs.

## Results

### Study design and data collection

The study was conducted according to the guidelines of the Declaration of Helsinki and approved by the Scientific Board of Istituto Tumori ‘Giovanni Paolo II’, Bari, Italy- prot. 17729/2020. Then, this study was determined by the Scientific Board to not require written consent from subjects, as it is retrospective and involves minimal risk.

A binary classification task was developed to give the prediction of 1-year disease-free survival in melanoma patients starting from the analysis of whole slide images (WSIs) representing cutaneous melanoma. Hence, patients were discerned in disease free (DF) and non-disease-free (non-DF) cases.

In the current paper, the experimental data referred to two diverse cohorts of patients were analysed. The predicted model was designed and firstly validated by exploiting data from CPTAC-CM public database^26^, which is part of The Cancer Imaging Archive (TCIA)^27^. The database contains cases of 49 patients with 1-year follow-up, counting 35 DF patients and 14 non-DF patients. Haematoxylin and eosin (H&E) images of cutaneous melanoma and some clinical data, such as gender, tumor site, pathological stage (stage), pathologic staging primary tumor (T), age, were downloaded from the CPTAC-CM online website. With respect to the pathologic staging of regional lymph nodes (N), the patients were distributed as: 34.9% as N0, 6.8% as N1, 9.3% as N2, 9.3% as N3 and 39.7 as NX, where NX means that it was not possible to determine whether the tumor has spread to the lymph nodes. Only patients with stage I–III melanomas as well as patients whose histological images were judged as good quality by our pathologist experts were considered as eligible for our analysis. Thus, a final cohort of 43 patients, out of which 31 DF patients and 12 non-DF patients, were retained. The cancer staging was evaluated according to either the seventh edition or the eighth edition of AJCC classification (36 patients and 7 patients, respectively). Anyway, this difference does not create bias in our analysis, since the two editions differ among each other for the evaluation of the only patients with T1 melanoma^28^. Clinical data characteristics of the 43 patients from CPTAC-CM dataset provided in Table 1.

**Table 1:** Clinical data referred to CPTAC-CM public dataset.

Characteristic	Distribution
**Outcome**	
DF cases (%)	31 (72.1%)
non-DF cases (%)	12 (27.9%)
**Gender**	
Male (%)	21 (48.8%)
Female (%)	22 (51.2%)
**Tumor site**	
Trunk (%)	20 (46.6%)
Palms and (%)	1 (2.3%)
Extremities (abs.; %)	11 (25.6%)
Head & neck (%)	1 (2.3%)
Lymph nodes (%)	5 (11.6%)
Other (%)	5 (11.6%)
**Stage**	
I (abs.; %)	6 (14.0%)
II (abs.; %)	27 (62.7%)
III (abs.; %)	10 (23.3%)
**T**	
T0 (abs.; %)	2 (4.7%)
T1 (abs.; %)	3 (6.8%)
T2 (abs.; %)	10 (23.3%)
T3 (abs.; %)	6 (14.0%)
T4 (abs.; %)	22 (51.2%)
**Age**	
Median (std.)	64.0 (14.9)

For categorical variables, percentage (%) counts are reported. For continuous values, the median and standard deviation (sd.) values are indicated.

The abbreviation *DF* stands for disease free. Among the five patients for which the tumor site has been labelled as ‘Other’, more detailed specifics about the tumor site were available online. For two of these patients, the primary site was specified to be unknown. The other three tumor sites were the left shoulder, the right shoulder and the IV toe of the left foot, respectively.

The model was further tested on a validation cohort of 11 cutaneous melanoma patients, out of which 8 DF and 3 non-DF at 1-year follow-up, whose data, consisting in H&E slides as well as clinical data were provided by our Institute. The WSIs were acquired by Camera Cmos DFK, Nikon, 33UX183 and saved as pyramidal images. Clinical data were summarized in Table 2.

**Table 2:** Clinical data referred to the validation cohort of patients.

Characteristic	Distribution
**Outcome**	
DF cases (%)	8 (72.7%)
non-DF cases (%)	3 (27.3%)
**Gender**	
Male (%)	3 (27.3%)
Female (%)	8 (72.7%)
**Tumor site**	
Trunk (%)	5 (45.5%)
Extremities (%)	3 27.3%)
Head & neck (%)	1 (9.1%)
Other (%)	2 (18.2%)
**Stage**	
I (%)	2 (18.2%)
II (%)	9 (81.8%)
**T**	
T2 (%)	2 (18.2%)
T3 (%)	4 (36.3%)
T4 (%)	5 (45.5%)
**Age**	
Median (std.)	56.0 (13.2)

For categorical variables, percentage (%) counts are reported. For continuous values, the median and standard deviation (sd.) values are indicated. The tumor site labelled as ‘Other’ was the gluteus muscle for both patients.

### Disease-free survival prediction

The designing and the first validation of the proposed predictive model was performed on data related to patients of the CPTAC-CM dataset. The identification of quantitative imaging biomarkers was executed by means of the three CNNs described in the Methods section. Basically, the WSIs were manually annotated by expert histopathologists of our Institute to identify a Region Of Interest (ROI), that were automatically divided in tiles. As detailed described in Methods Section, only tiles with high cell density were retained and then partitioned in crops of dimension equal to one-fourth of the tile size. The three deep models built in correspondence of the three CNNs were firstly performed separately and then ensembled together. In Table 3, the median AUC values reached by the three deep models firstly at crop level, and then at WSI level, are compared. To pass from crop level to WSI level, a vote score thresholding procedure based on the definition of a unique classification score for the WSI as the 75th percentile value of the distribution of the classification scores related to crops was implemented (*see* “Methods”). A median AUC value of 50.5%, 56.2% and 54.8% was achieved at crop level by *ResSVM*, *DenseSVM*, *InceptionSVM*, respectively. The implementation of the vote score thresholding procedure to obtain a unique AUC value per WSI and hence per patient led to an AUC improvement up to 10% for each of the three deep models: a median AUC value of 64.7%, 64.8% and 64.9% was returned by *ResSVM*, *DenseSVM*, *InceptionSVM*, respectively. The performances were balanced among the models, thus pointing out the robustness of the proposed data analysis pipeline.

**Table 3:** Comparison of the median percentage AUC values achieved on both crops and entire WSIs by means of the three deep models, ResSVM, DenseSVM and InceptionSVM.

	Model		
	*ResSVM*	*DenseSVM*	*InceptionSVM*
AUC on crops (%)	50.5 [49.8 55.0]	56.2 [48.5 64.7]	54.8 [51.7 56.4]
AUC on WSIs (%)	64.7 [58.6 66.4]	64.8 [63.6 69.5]	64.9 [64.1 69.0]

The three deep models were overcome by the ensemble model, *DeepSVM*, which reached a median AUC value of 69.5%, as shown in Table 3.

**Figure 1:**
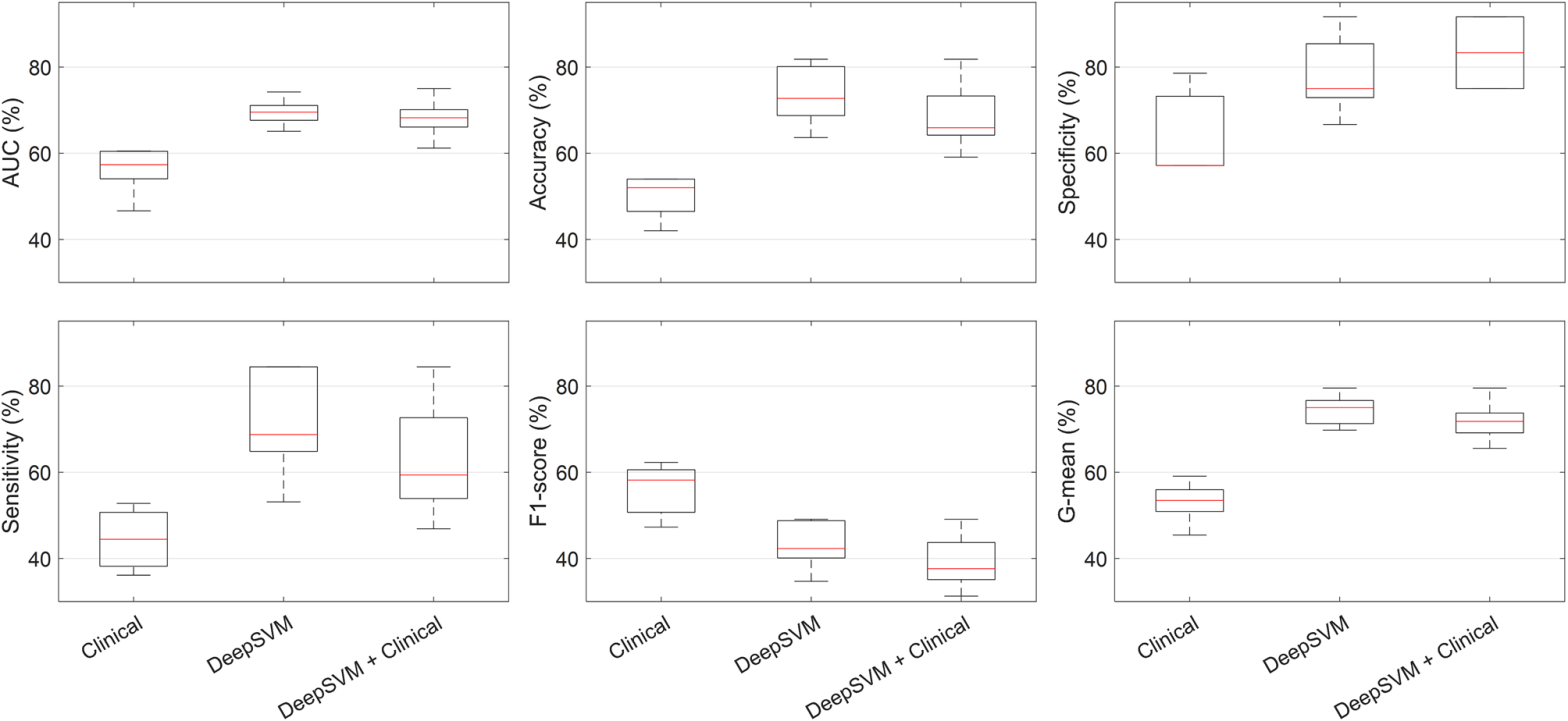
Boxplots of the performances of the 1-year disease-free survival predictive models in terms of percentage AUC, accuracy, sensitivity, specificity, F1-score and G-mean. The results are evaluated by implementing a fivefold cross validation scheme for five rounds on the CPTAC-CM public dataset.

Performing an association test between 1-year disease-free survival outcome and each clinical factor no significant associations were recognized for both public and validation cohorts of patients. By following the data analysis pipeline, we then designed a SVM classifier, which exploits all the clinical variables at disposal (*see* Table 1) according to a fivefold cross validation scheme on the CPTAC-CM public dataset. In our experimental analysis, we attempted to implement two feature selection algorithms: Random Forest which computes Gini impurity to identify the most important features^29^; forward sequential feature selection algorithm^30^, which selected features over the training set during cross validation until there is no improvement in prediction of an SVM classifier over the same training set. However, no significant improvement in performances were obtained. For this reason, we reported only the results achieved by the model using all clinical variables. Indicated as *Clinical* in Fig. 1, this model reached a median AUC value of 57.3%, a median accuracy value of 52.0%, a median sensitivity value of 44.4%, a median specificity value of 57.1%, a median F1-score value of 58.2%, and a median Geometric mean (G-mean) value of 53.5%.

With respect to the *Clinical* model, *DeepSVM* model led to great improvement for all the performance evaluation metrics except for F1-score (*see* Fig. 1): a median AUC value of 69.5%, a median accuracy value of 72.7%, a median sensitivity value of 68.8%, a median specificity value of 75.0%, a median F1-score value of 42.3%, and a median G-mean value of 75% were obtained, respectively. The sensitivity and specificity values were well balanced. The *DeepSVM* + *clinical* model, defined by combining the scores achieved by *DeepSVM* and *Clinical* separately via a soft voting technique, did not lead to an overall improvement of the results with respect to the *DeepSVM* model. The only metric which increased was the median specificity value (83.3%), but at the expense of the median sensitivity value (59.4%). However, if compared with the *Clinical* model alone, the *DeepSVM* + *clinical* model returned better performance for all the evaluation metrics except for F1-score (*see* Fig. 1).

**Figure 2:**
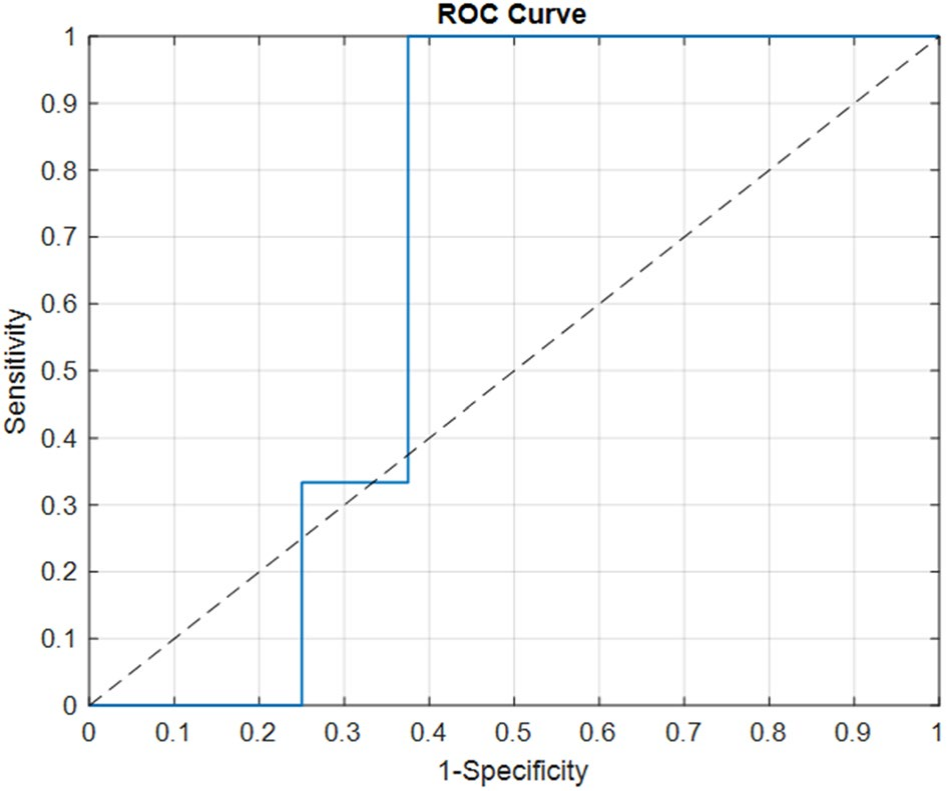
ROC Curve related to the *DeepSVM* model on the validation cohort. The dotted line represents the random guess.

The model which showed the best performances was those obtained by exploiting the image information alone, i.e. *DeepSVM*. This best performing model was finally tested on the validation cohort of patients recruited from our Institute, reaching an AUC value of 66.7%, an accuracy value of 72.7%, a sensitivity value of 100%, a specificity value of 62.5%, a F1-score value of 66.6%, a G-mean value of 79.1%. The resulting the Receiver Operating Characteristic (ROC) curve is represented in Fig. 2. The achieved results demonstrated how the proposed model was quite robust and generalizable.

## Discussion

The post-surgery evaluation of disease-free survival outcome has gained increasing attention for management of melanoma patients in consequence of the continuous progress in defining even more suitable therapies during the last few years. Whether WSI analysis can improve the prediction of prognostic tasks related to melanoma with respect to considering clinical data alone is currently under scrutiny within the scientific community^23–25,31,32^. As example, Peng et al.^31^ developed lasso Cox prediction models based on the integration of clinical variables, gene signature and WSI features to predict recurrence-free survival in melanoma patients. The integration of WSI features with baseline clinical variable improved performance obtained by using clinical variable only. However, they made use of handcrafted features, that, as demonstrated elsewhere^33^, can be affected by human bias. The advent of deep learning has attracted great interest of both clinical and technical figures operating together in multidisciplinary teams of cancer centers, since it has opened the path to the possibility of performing an automatic feature extraction from raw images without human intervention. The extracted features were recognized as able to discern complex structure underlying image texture, which are usually hidden to human eyes^15^. In this study, we wanted to make a contribution on the development of a cost-effective prognostic model by integrating clinical variables with quantitative WSI information automatically extracted via deep learning. We developed a deep learning-based model which make use of features extracted by transfer learning with the aim of learning prognostic biomarkers from WSIs to predict 1-year disease-free survival in stage I–III cutaneous melanoma patients. To the best of our knowledge, this is the first work which exploits transfer learning for this prognostic task. Transfer learning allows to handle the limited size of the datasets at disposal in this study. Small datasets are very common in clinical studies due to the complexity and high costs of patient data collection. It is well-known how CNNs trained and validated on small datasets tends to lose its prediction power, being more prone to overfitting and unstable predictions^34^. The application of transfer learning combined with cross validation and ensemble model strategies enables to overcome these technical issues, also making the achieved results more generalizable. As proof of concept, in this paper, we firstly designed and validated the model on a public dataset, and, afterwards, we tested the robustness and generalizability of the model on a cohort of melanoma patients recruited from our Institute. Basically, we made use of pre-trained networks as feature extractors (transfer learning) only and SVMs as standard classifiers. The combination of clinical and imaging data used by *DeepSVM* + *Clinical* model led to a clear improvement in performances if compared with the model *Clinical* using clinical variables only: the reached AUC values were 68.2% and 57.3%, respectively. However, clinical variables did not add significant information when added to the imaging features. Indeed, the best predictive classification performances were obtained in terms of median AUC and accuracy with values of 69.5% and 72.7%, respectively, when the only quantitative imaging biomarkers extracted by CNNs were evaluated. The abovementioned results were achieved on the CPTAC-CM public dataset, remaining comparable when the model was tested on the validation cohort of melanoma patients (AUC value of 66.7% and accuracy value of 72.7%, respectively).

A fundamental peculiarity of the proposed model is the automatic identification of quantitative imaging information from the raw WSIs directly. In other words, we used a computerized system to automatically extract information that are usually evaluated manually and visually by pathologists. Our model was able to automatically capture fine tumor or lymphocytic infiltrate characteristics, such as morphology of tumor nuclei as well as density distribution of lymphocytes, that are well-known to be associated with metastasis and survival outcomes^35,36^, and then with the disease-free survival.

The limitation of this study is represented by the relatively small size of the analysed datasets. The generalizability of the proposed model should be further validated on wider cohorts of patients. The next step of our research work will be to train the algorithm on WSIs of patients recruited from our Institute in the hypothesis of improving the predicting performances and then validate the robustness in multi-centric studies. Here, we have extracted high-dimensional features, which refer to global cues of an image, such as shapes or entire objects. We are planning to add key low-dimensional features, i.e. features that are related to local image characteristics, to the developed model.

Beyond the validation on larger datasets, in future extensions of our work, eXplainable Artificial Intelligence (XAI) models^37^ will be integrated to make the decisions achieved by the proposed model as more intelligible to humans. The comprehension and trust of the outcomes created by artificial intelligence algorithms is essential to an easier applicability of artificial intelligence in clinical practice. In our case, the optimization of our model through XAI could lead to both effective and low-cost prognostic model to be used to manage the care of melanoma patients in routine clinical practice.

Finally, the present study proposed an artificial intelligence model to determine which cutaneous melanoma patients could show 1-year disease-free survival thanks to a direct identification of quantitative imaging biomarkers from WSIs. The promising results achieved in this preliminary work suggest how our proposal, after further validations of wider cohorts of patients as well as technical refinement, has the potential to fulfil the predictive task with great improvement in the melanoma patient management in terms of time and costs, also representing a complementary tool with respect to the current genetic and manual immunohistochemistry methods.

## Methods

### Image pre-processing

**Figure 3:**
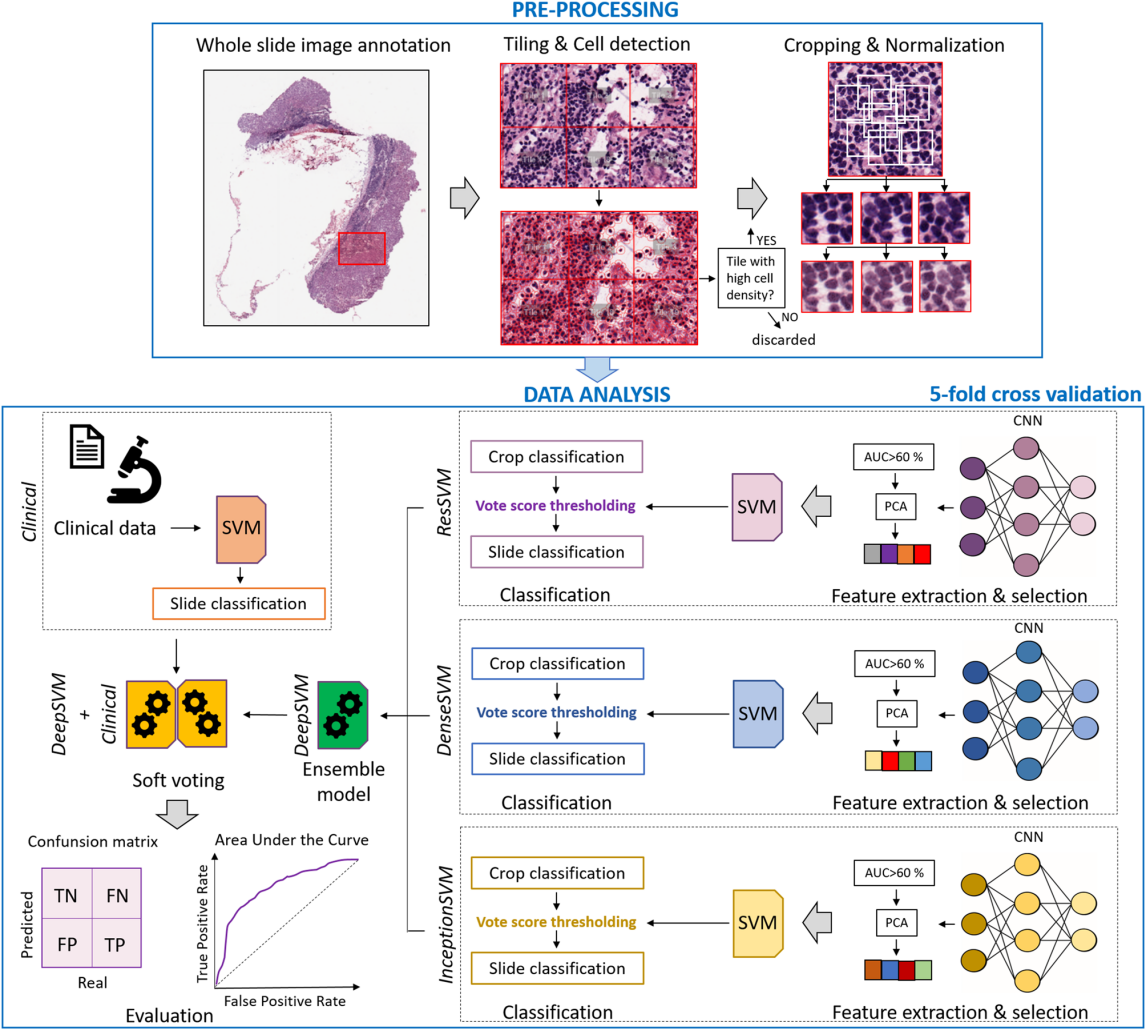
Workflow of the proposed approach. Image pre-processing was firstly performed: WSIs were annotated by expert pathologists; the identified Region of Interests (ROIs), one per WSI, were tessellated into tiles of 224 × 224 pixels; cell detection was performed and only tiles with high cell density were retained and then divided into low-dimensional crops, which were finally colour-normalized. Data-analysis was then performed: crops were taken in input by three pre-trained CNNs. Each CNN extracted thousands of imaging features, later undergoing to a feature selection process; the selected features were employed to build a SVM classifier, which expressed a decision (DF or non-DF) firstly at crop level and then at slide level through the implementation of a vote score thresholding. In correspondence of the three CNNs, three models, called *ResSVM*, *DenseSVM* and *InceptionSVM* were defined. An ensemble model, named *DeepSVM*, was designed by combining the classification scores of the three models. A model, named *Clinical*, which took in input clinical data and used a SVM classifier to give in output classification scores at WSI level, was defined. A soft-voting procedure was implemented to combine the classification scores of *DeepSVM* and *Clinical* at WSI level and then at patient level*.* The final model was called *DeepSVM* + *Clinical.* Predictive performances were assessed by standard evaluation metrics.

Histopathological images are pyramidal images and hence hard to be taken in input to an artificial intelligence algorithm directly. Hence, an image pre-processing phase was performed, as shown in the upper panel of Fig. 3. Two expert histopathologists of our Institute selected and then annotated the most representative WSI per patient to mark a Region Of Interest (ROI). Each ROI was split into tiles with 224 × 224 pixels at 20 × magnification using QuPath open-source software^38^. An automated cell detection to identify both tumor cells and lymphocytes was run on each tile. Only tiles with high cell density were retained. Cell density was computed as the ratio between the number of cells within the tile and the tile area. The distribution of cell density referred to tiles of a same ROI were then defined. A tile was considered as containing a high cell density, i.e. a high cell content information, if the related cell density exceeded the 90th percentile of the distribution. All the other tiles were discarded (*see* Fig. 3). The retained tiles were then partitioned in crops of dimension equal to one-fourth of the tile size. The centres of the crops were obtained as points randomly sampled from a 2D Gaussian distribution centred on the tile. A maximum number of 50 crops per tile were generated. The number of crops varied from tile to tile since only crops with less than 25% background pixels (Luma > 170) were retained for further analysis. A total of 12,575 crops were extracted from WSIs related to the CPTAC-CM dataset, while an amount of 4011 crops were lastly obtained from WSIs related to the validation cohort referred to our Institute. Finally, the colour of each crop was normalized by a standard WSI normalization algorithm, known as Macenko’s method^39^, to overcome many inconsistencies during staining process of the histological slides due to either different stain manufacturers or different staining procedures or even different storage times.

### Data analysis pipeline

The data analysis pipeline following image pre-processing is depicted in the bottom panel of Fig. 3. We used commonly cutting-edge pre-trained CNNs implemented in MATLAB R2019a (MathWorks, Inc., Natick, MA, USA) software, such as ResNet50^40^, DenseNet201^41^, InceptionV3^42^, to extract high-dimensional imaging features from crops. ResNet50 model^40^ is a 50 layers deep CNN which makes possible to train much deeper networks maintaining compelling performances by stacking residual blocks on top of each other. All crops were resized as 224 × 224 size images to be taken in input to ResNet50. A total of 2048 features were finally extracted. DenseNet201^41^ model is composed by layers receiving additional inputs from all preceding layers and passing their feature-maps to all subsequent layers. All crops were resized as 224 × 224 size images to be taken in input to DenseNet201. A total of 1920 features were finally extracted. InceptionV3^42^ has an architectural design with repeated components called inception modules. All crops were resized as 299 × 299 size images to be taken in input to InceptionV3. A total of 2048 features were finally extracted. The weights of the pre-trained CNNs referred to the training on ImageNet dataset. The networks have acquired knowledge from a huge number of natural (non-medical) images of the ImageNet dataset during the training phase that, in this paper, has been transferred on never unseen images (WSIs). In our study, only the labels of crops, inherited by the WSIs to which they belong (DF cases vs non-DF cases) were needed for the classification phase (SVM classifiers, *see* later), while transfer learning was entirely performed using an unsupervised method.

In correspondence of features extracted from the three CNNs, three deep models, called *ResSVM*, *DenseSVM* and *InceptionSVM*, respectively, were designed.

The three deep models shared the same backbone architecture, which is described in the following. After feature extraction, the initial public cohort of 43 patients was divided in turn into training and test sets, according to a fivefold cross validation procedure for 5 rounds. In this manner, all the crops associated to the WSI of one patient were part either of the training set or the test set depending on whether the patient was assigned to the training set or the test set, respectively. We implemented a nested waterfall feature selection technique consisting of two basic feature selection methods. First, features were selected according to their statistical significance which was assessed through the computation of Area Under the receiver operating Curve (AUC), which accounts for the general discriminative capability of each feature with respect to a binary classification problem: this metric takes percentage values ranging from 50 to 100%, indicating random guess and perfect discriminatory ability, respectively^43^. Features whose AUC value was less than 60% were dropped. Afterwards, principal component analysis (PCA)^44^ based on the explained variance criterion was performed to further reduce the number of features selected by the first algorithm. The first *p* components were chosen according to the percentage of variance explained (we fixed a threshold of 80%) by total selected components and later taken in input by a Support Vector Machine (SVM) classifier with radial basis kernel function^45^. A first decision was returned at crop level by the classifier: a classification score ranging from 0 to 1 was assigned to each crop separately. To obtain a single classification score for each WSI and then for each individual patient, a vote score thresholding procedure was implemented: the distribution of the scores attributed by the classifier to each crop related to a same WSI was defined and the classification score corresponding to the 75th percentile value was assigned as the final classification score to the WSI and thus to the patient to which the WSI corresponded. Hence, three classification scores were assigned to each individual patient, each one in correspondence of each of the three deep models. These three classification scores were combined according to an ensemble procedure: if the classification scores returned by at least two out of three deep models exceeded the value *th* = 0.28, that corresponded to the ratio of DF patients over the total number of the sample population, the maximum score was assigned as a final score. Conversely, if the classification scores returned by at least two out of three deep models was less than *th*, the minimum score was attributed as final score. In the following, we refer to this model as *DeepSVM*.

As a last step of analysis, a SVM classifier exploiting the clinical features summarized in Table 1 was designed. The model, called *Clinical*, returned a classification score for each individual patient.

Finally, a soft voting technique, consisting of averaging the classification scores of diverse models, was implemented to combine the scores obtained by *DeepSVM* and *Clinical*. Thus, a new model with the name *DeepSVM* + *Clinical* was designed. The model between *DeepSVM and DeepSVM* + *Clinical* model*,* which returned the best predictive performances on the CPTAC-CM public dataset was further validated by using the 43 patients of the CPTAC-CM public dataset as training set and the validation cohort of 11 patients recruited from our Institute as test set.

### Statistical analysis and performance evaluation

The association between each clinical characteristic and disease-free survival was evaluated by means of statistical tests on overall dataset: Wilcoxon-Mann-Whitney test^46^ was used for continuous features, whereas Chi Squared test^47^ was employed for those features measured on an ordinal scale. A result was considered statistically significant when the p-value was less than 0.05.

The predictive performances of the developed models were evaluated in terms of AUC and, once the optimal threshold was identified by Youden’s index on ROC curve^48^, standard metrics, such as accuracy, sensitivity, and specificity were also computed:

$${\text{Accuracy }} = \, \left( {{\text{TP }} + {\text{ TN}}} \right)/ \, \left( {{\text{TP }} + {\text{ TN }} + {\text{ FP }} + {\text{ FN}}} \right),$$

$${\text{Sensitivity }} = {\text{ TP}}/ \, \left( {{\text{TP }} + {\text{ FN}}} \right),$$

$${\text{Specificity }} = {\text{ TN}}/ \, \left( {{\text{TN }} + {\text{ FP}}} \right),$$

where TP and TN stand for True Positive (number of non-DF cases correctly classified) and True Negative (number of DF cases correctly classified), while FP (number of DF cases identified as non-DF cases) and FN (number of non-DF cases identified as DF cases are False Positive and False Negative ones, respectively. In this paper, since we solved a binary classification problem (DF cases vs non-DF cases) on imbalanced data (*see* Tables 1, 2), we also computed two other metrics, i.e. F1-score and Geometric mean (G-mean), that have been suggested as suitable for practitioners to determine an appropriate performance measure in the case of analysis of imbalanced datasets^49^.

The F1-score works well for imbalanced datasets since the relative contribution of precision and sensitivity are computed as equal:

$${\text{F1}} - {\text{score }} = { 2 }* \, \left( {{\text{sensitivity}}*{\text{ precision}}} \right) \, /\left( {{\text{sensitivity }}*{\text{ precision}}} \right),$$

with precision = TP/(TP + FP).

The Geometric Mean (G-Mean) measures the balance between classification performances on both the classes, thus avoiding overfitting the class with the major number of subjects and underfitting the class with the minor number of subjects:

$$\mathrm{G}-\mathrm{mean }= \sqrt{\mathrm{Sensitivity}*\mathrm{Specificity}}.$$

### Ethics approval and consent to participate

The study was conducted according to the guidelines of the Declaration of Helsinki and approved by the Scientific Board of Istituto Tumori ‘Giovanni Paolo II’, Bari, Italy- prot 17729/2020. The authors affiliated to Istituto Tumori “Giovanni Paolo II”, IRCCS, Bari are responsible for the views expressed in this article, which do not necessarily represent the ones of the Institute.

## Data Availability

Public data used in this publication were generated by the National Cancer Institute Clinical Proteomic Tumor Analysis Consortium (CPTAC)**:** https://wiki.cancerimagingarchive.net/display/Public/CPTAC-CM#33948224bcab02c187174a288dbcbf95d26179e8**.** Data of patients recruited from Istituto Tumori “Giovanni Paolo II” are at disposal under request from the corresponding author. QuPath source codes as well as MATLAB codes of the methodology could be found at the following link: https://github.com/mcomes92/melanoma.

## References

[1] Han SS, Kim MS, Lim W (2018). Classification of the clinical images for benign and malignant cutaneous tumors using a deep learning algorithm. J Investig. Dermatol..

[2] Ali Z, Yousaf N, Larkin J (2013). Melanoma epidemiology, biology and prognosis. Eur. J. Cancer Suppl..

[3] https://www.cancer.org/cancer/melanoma-skin-cancer/about/key-statistics.html#:~:text=Cancer%20of%20the%20skin%20is,majority%20of%20skin%20cancer%20deaths. Accessed 7 August 2022.

[4] Robert C, Thomas L, Bondarenko I (2011). Ipilimumab plus dacarbazine for previously untreated metastatic melanoma. N. Engl. J. Med..

[5] Ascierto PA, Borgognoni L, Botti G (2019). New paradigm for stage III melanoma: From surgery to adjuvant treatment. J. Transl. Med..

[6] Guida M, Pisconte S, Colucci G (2012). Metastatic melanoma: The new era of targeted therapy. Expert Opin. Ther. Targets.

[7] Johnson DB, Chandra S, Sosman JA (2018). immune checkpoint inhibitor toxicity. JAMA—J. Am. Med. Assoc..

[8] Gordan L, Blazer M, Saundankar V (2019). Cost differential of immuno-oncology therapy delivered at community versus hospital clinics. Am. J. Manag. Care.

[9] Hyams DM, Cook RW, Buzaid AC (2019). Identification of risk in cutaneous melanoma patients: Prognostic and predictive markers. J. Surg. Oncol..

[10] Trinidad CM, Torres-Cabala CA, Curry JL (2019). Update on eighth edition American Joint Committee on Cancer classification for cutaneous melanoma and overview of potential pitfalls in histological examination of staging parameters. J. Clin. Pathol..

[11] Renner P, Torzewski M, Zeman F (2017). Increasing morbidity with extent of lymphadenectomy for primary malignant melanoma. Lymphat. Res. Biol..

[12] Gerami P, Cook RW, Russell MC (2015). Gene expression profiling for molecular staging of cutaneous melanoma in patients undergoing sentinel lymph node biopsy. J. Am. Acad. Dermatol..

[13] Sivendran S, Chang R, Pham L (2014). Dissection of immune gene networks in primary melanoma tumors critical for antitumor surveillance of patients with stage II-III resectable disease. J. Investig. Dermatol..

[14] Farahani N, Parwani AV, Pantanowitz L (2015). Whole slide imaging in pathology: Advantages, limitations and emerging perspectives. Pathol. Lab Med. Int..

[15] LeCun Y, Bengio Y, Hinton G (2015). Deep learning. Nature.

[16] Bellotti R, Bagnasco S, Bottigli U (2004). The MAGIC-5 project: Medical applications on a grid infrastructure connection. IEEE Nucl. Sci. Symp. Conf. Rec..

[17] Bellotti R, De Carlo F, De Tommaso M, Sciruicchio V (2004). Topographic classification of EEG patterns in Huntington’s disease. Neurol. Clin. Neurophysiol. NCN.

[18] Uchida S, Ide S, Iwana BK, Zhu A (2016). A further step to perfect accuracy by training CNN with larger data. Proc. Int. Conf. Front. Handwrit. Recognit. ICFHR.

[19] Comes MC, Fanizzi A, Bove S (2021). Early prediction of neoadjuvant chemotherapy response by exploiting a transfer learning approach on breast DCE-MRIs. Sci. Rep..

[20] Comes MC (2021). Early prediction of breast cancer recurrence for patients treated with neoadjuvant chemotherapy: A transfer learning approach on DCE-MRIs. Cancers.

[21] Hekler A, Utikal JS, Enk AH (2019). Deep learning outperformed 11 pathologists in the classification of histopathological melanoma images. Eur. J. Cancer.

[22] Hekler A, Utikal JS, Enk AH (2019). Pathologist-level classification of histopathological melanoma images with deep neural networks. Eur. J. Cancer.

[23] Brinker TJ, Kiehl L, Schmitt M (2021). Deep learning approach to predict sentinel lymph node status directly from routine histology of primary melanoma tumours. Eur. J. Cancer.

[24] Hu J, Cui C, Yang W (2021). Using deep learning to predict anti-PD-1 response in melanoma and lung cancer patients from histopathology images. Transl. Oncol..

[25] Kulkarni PM, Robinson EJ, Pradhan JS (2020). Deep learning based on standard H&E images of primary melanoma tumors identifies patients at risk for visceral recurrence and death. Clin. Cancer Res..

[26] National Cancer Institute Clinical Proteomic Tumor Analysis Consortium (CPTAC) Radiology Data from the Clinical Proteomic Tumor Analysis Consortium Cutaneous Melanoma [CPTAC-CM] collection. *The Cancer Imaging Archive*. https://www.cancerimagingarchive.net (2018).

[27] Clark K, Vendt B, Smith K, Freymann J, Kirby J, Koppel P, Moore S, Phillips S, Maffitt D, Pringle M, Tarbox L, Prior F (2013). The Cancer Imaging Archive (TCIA): Maintaining and Operating a Public Information Repository. J. Digit. Imaging.

[28] Teterycz P, Ługowska I, Koseła-Paterczyk H, Rutkowski P (2019). Comparison of seventh and eighth edition of AJCC staging system in melanomas at locoregional stage. World J Surg Oncol.

[29] Breiman L (2001). Random forests. Mach. Learn..

[30] Rückstieß T., Osendorfer C., Van Der Smagt P. Sequential feature selection for classification. Lect Notes Comput Sci (including Subser Lect Notes Artif Intell Lect Notes Bioinformatics) 7106 LNAI 132–141 10.1007/978-3-642-25832-9_14 (2011).

[31] Peng Y, Chu Y, Chen Z (2020). Combining texture features of whole slide images improves prognostic prediction of recurrence-free survival for cutaneous melanoma patients. World J. Surg. Oncol..

[32] Guida M, Strippoli S, Maule M (2021). Immune checkpoint inhibitor associated vitiligo and its impact on survival in patients with metastatic melanoma: An Italian melanoma intergroup study. ESMO Open.

[33] Ordóñez FJ, Roggen D (2016). Deep convolutional and LSTM recurrent neural networks for multimodal wearable activity recognition. Sensors.

[34] Shaikhina T, Khovanova NA (2017). Handling limited datasets with neural networks in medical applications: A small-data approach. Artif. Intell. Med..

[35] Pastorfide GC, Kibbi A-G, de Roa AL (1992). Image analysis of stage 1 melanoma (1.00–2.50 mm): Lymphocytic infiltrates related to metastasis and survival. J. Cutan. Pathol..

[36] Ralfkiaer E, Hou-Jensen K, Gatter KC (1987). Immunohistological analysis of the lymphoid infiltrate in cutaneous malignant melanomas. Virchows Arch. A Pathol. Anat. Histopathol..

[37] Barredo Arrieta A, Díaz-Rodríguez N, Del Ser J (2020). Explainable explainable artificial intelligence (XAI): Concepts, taxonomies, opportunities and challenges toward responsible AI. Inform. Fusion.

[38] Bankhead P, Loughrey MB, Fernández JA (2017). QuPath: Open source software for digital pathology image analysis. Sci. Rep..

[39] Macenko M., Niethammer M, Marron J.S. et al. A method for normalizing histology slides for quantitative analysis 3 Statistics and Operations Research, 4 Lineberger Comprehensive Cancer Center, 5 Renaissance Computing Institute, 6 Pathology and Laboratory Medicine, 7 Dermatology University of Nor. IEEE Int Symp Biomed Imaging 1107–1110 (2009).

[40] He K. Deep Residual Learning for Image Recognition ResNet @ ILSVRC & COCO 2015 Competitions 1–9 (2015).

[41] Huang G., Liu Z., Van Der Maaten L., Weinberger K.Q. Densely connected convolutional networks. In *Proc. 30th IEEE Conf Comput Vis Pattern Recognition, CVPR 2017 2017-Jan* 2261–2269 10.1109/CVPR.2017.243 (2017).

[42] Szegedy C., Vanhoucke V., Ioffe S. et al. Rethinking the inception architecture for computer vision. In *Proc. IEEE Comput Soc Conf Comput Vis Pattern Recognit 2016-Dec* 2818–2826 10.1109/CVPR.2016.308 (2016).

[43] Fawcett T (2006). An introduction to ROC analysis. Pattern Recognit. Lett..

[44] Zhou P, Lu C, Lin Z (2021). Tensor principal component analysis. Tensors Data Process Theory Methods Appl..

[45] Burges CJ (1998). A tutorial on support vector machines for pattern recognition. Data Min. Knowl. Discov..

[46] Mann HB, Whitney DR (1947). On a test of whether one of two random variables is stochastically larger larger than the other. Ann. Math. Stat..

[47] Pandis N (2016). The chi-square test. Am. J. Orthod. Dentofac. Orthop..

[48] Youden WJ (1950). Index for rating diagnostic tests. Cancer.

[49] Akosa JS (2017). Predictive accuracy: A misleading performance measure for highly imbalanced data. SAS Glob. Forum.

